# Simultaneous Removal of Arsenic and Manganese from Synthetic Aqueous Solutions Using Polymer Gel Composites

**DOI:** 10.3390/nano11041032

**Published:** 2021-04-18

**Authors:** Syed Ragib Safi, Takehiko Gotoh

**Affiliations:** Department of Chemical Engineering, Hiroshima University, 1-4-1 Kagamiyama, Higashi Hiroshima, Hiroshima 739-8527, Japan; d196289@hiroshima-u.ac.jp

**Keywords:** polymer gel, arsenic, manganese, water, adsorption, cationic gel

## Abstract

The groundwater in approximately 50% of the Bangladesh landmass contains Mn concentrations greater than the limit prescribed by the WHO drinking water guidelines. Although studies have suggested that γ-FeOOH can effectively remove Mn from water, its practicability has not been investigated, considering that the additional processes required to separate the adsorbents and precipitates are not environment-friendly. To improve the efficiency of adsorptive Mn-removal under natural conditions, we employed a cationic polymer gel composite, *N*,*N*’-Dimethylaminopropyl acrylamide, methyl chloride quaternary (DMAPAAQ) loaded with iron hydroxide (DMAPAAQ + FeOOH), and a non-ionic polymer gel composite, *N*,*N*’-Dimethylacrylamide (DMAA) loaded with iron hydroxide (DMAA + FeOOH). DMAPAAQ + FeOOH exhibited a higher As removal efficiency under natural conditions while being environment-friendly. Our results suggest that the higher efficiency of the cationic gel composite is owed to the higher γ-FeOOH content in its gel structure. The maximum adsorption of Mn by DMAPAAQ + FeOOH was 39.02 mg/g. Furthermore, the presence of As did not influence the adsorption of Mn on the DMAPAAQ + FeOOH gel composite and vice versa. DMAPAAQ adsorbed As and the γ-FeOOH particles simultaneously adsorbed Mn. Our findings can serve as a basis for the simultaneous removal of contaminants such as As, Mn, Cr, and Cd.

## 1. Introduction

Manganese (Mn) is a known mutagen whose accumulation may cause hepatic encephalopathy. Consumption of Mn through drinking water may cause neurological damage. The WHO has set a limit of 0.5 mg/L for Mn in drinking water because of its neurotoxicity and other detrimental effects in humans and other animals in Japan and Greece [1].

Following arsenic (As), Mn is one of the most commonly-found contaminants in most rivers and groundwater in Bangladesh [2]. Approximately 50% of the area of Bangladesh contains groundwater having Mn concentrations greater than suggested by the World Health Organization (WHO) health-based drinking water guidelines. The maximum concentration of Mn in groundwaters in Bangladesh was found to be 2.0 mg/L, whereas the WHO approves a maximum of 0.5 mg/L of Mn in drinking water. In approximately 35% of the samples collected by Frisbie et al., the WHO drinking water guidelines were exceeded for As and Mn [1]. Besides, the maximum concentration of Mn in industrial wastewater in Bangladesh was found to be as high as 5.0 mg/L [3,4]. In addition, As and Mn are among the most critical pollutants of drinking water in the South African region [5]. The coexistence of As and Mn in groundwater has been reported earlier [6]. Therefore, the simultaneous removal of As and Mn needs to be studied.

Many studies have been conducted on the removal of As and a few have focused on the removal of Mn. However, studies on simultaneous removal of As and Mn are rare because it is difficult to remove anionic and cationic components using a single adsorbent. Although As and Mn have been removed separately or exclusively [7,8], their simultaneous adsorption has not been conducted to date. Two previous studies have reported the removal of As, Mn, and Fe [6,9] and one study developed a treatment system using coagulation and filtration for the simultaneous removal of As and Mn [10]. Although these three studies claimed to remove As, they removed only one oxidation state of arsenic, arsenite (As(III)). In these studies, As(III) was oxidised by the coexisting MnO_2_, resulting in the removal of As(III) and Mn from the initial solution. Furthermore, the influence of the precipitate formed due to the coexistence of As(III) and Mn on the removal efficiencies of the methods has not been investigated using response surface methodology (RSM), microorganisms in a biofilter and the sedimentation-filtration treatment process [10]. Furthermore, in solutions containing arsenate (As(V)) and As(III), the extent of simultaneous removal of As and Mn would differ, because As(V) is not oxidised by MnO_2_. Furthermore, no studies were found on the simultaneous removal of Mn and total As. Finally, the changes in the surface functional groups were not investigated using Fourier transform infrared (FTIR) spectroscopy, and the adsorption mechanisms were not confirmed in the aforementioned works. Therefore, we have conducted a pioneering study on the simultaneous removal of total As and Mn using a single polymer-based adsorbent, without requiring additional separation processes.

Previously, we reported the effective removal of As from groundwater using our novel adsorbent, *N*,*N*’-Dimethylaminopropyl acrylamide, methyl chloride quaternary (DMAPAAQ), loaded with γ-FeOOH (DMAPAAQ + FeOOH) [11,12,13]. It has been reported that γ-FeOOH effectively removes Mn [14]. However, the efficient removal of Mn was achieved at a high pH. Furthermore, the performance of γ-FeOOH at removing Mn from water under natural conditions was not evaluated. The impregnation of Fe in adsorbents such as pumice increased the adsorption of Mn by approximately 14 times [15]. Polymer-based adsorbents are effective at removing As in both of its oxidation states [16,17]. However, there is no prior study on using polymer-based adsorbents to adsorb Mn. Therefore, we investigated the Mn removal efficiency of an Fe-impregnated cationic polymer gel.

In this study, we investigated the adsorptive removal of Mn using our previously-developed gel, DMAPAAQ + FeOOH [11,12,13]. Additionally, we compared the Mn removal efficiency of DMAPAAQ + FeOOH gel to that of the non-ionic gel, *N*,*N*’-Dimethylacrylamide (DMAA) and non-ionic gel composite (DMAA + FeOOH). Finally, we conducted a simultaneous adsorption of As and Mn. Furthermore, we investigated the pH-sensitivity of the Mn adsorption, and used FTIR spectroscopy to examine whether Mn adheres to the surface functional group of the gel composite. Although various factors such as pH and temperature have significant effects on the removal of Mn [18], we conducted our experiments while maintaining natural conditions. The objective of this research is to effectively remove two of the most harmful elements in groundwater, As and Mn, simultaneously and under natural water conditions.

## 2. Materials and Methods

### 2.1. Materials

The monomer, *N*,*N*’-Dimethylaminopropyl acrylamide, methyl chloride quaternary (DMAPAAQ) (75% in H_2_O) was supplied by KJ Chemicals Corporation, Tokyo, Japan. The crosslinker, *N*,*N*’-methylene bisacrylamide (MBAA), and the As(III) source, sodium (meta)arsenite, were purchased from Sigma-Aldrich, St. Louis, MO, USA. The accelerator, sodium sulphite (Na_2_SO_3_), the manganese source, manganese sulphate pentahydrate MnSO_4_·5H_2_O, the arsenic(V) source, disodium hydrogen arsenate heptahydrate (Na_2_HAsO_4_·7H_2_0), the adsorbents, activated charcoal and silica gel, and ferric chloride (FeCl_3_) were purchased from Nacalai Tesque, Inc., Kyoto, Japan. Sodium hydroxide (NaOH) was purchased from Kishida Chemicals Corporation, Osaka, Japan. The monomer, *N*,*N*’-Dimethylacrylamide (DMAA) and 2-acrylamido 2-methyl propane sulfonic acid (AMPS) were purchased from TCI, Japan. The initiator, ammonium peroxodisulfate (APS) was purchased from Kanto Chemical Co. Inc., Tokyo, Japan.

### 2.2. Synthesis of the Polymer Gels and Composites

The polymer gels DMAPAAQ and DMAPAAQ + FeOOH were prepared by the free-radical polymerisation method described in our previous studies [11,12,13] and also in Tables S2 and S3. The amounts of the crosslinker, initiator, and accelerator were identical in the compositions of DMAPAAQ, DMAPAAQ + FeOOH, and DMAA + FeOOH. For the composites DMAPAAQ + FeOOH and DMAA + FeOOH, FeCl_3_ and NaOH were added to impregnate the iron components in the polymer gel structure [11,12,13]. The reason for adding NaOH was that NaOH reacted with FeCl_3_ during the polymerisation. Consequently, the majority of the FeOOH particles were produced inside the gel structure.

### 2.3. Adsorption Experiment

To perform the Mn-adsorption experiment, 20 mg of dried gel composite (γ-FeOOH, DMAPAAQ, DMAPAAQ + FeOOH, DMAA, activated charcoal, silica gel, and DMAA + FeOOH) was added to 40 mL of Mn or As solution in a small beaker and stirred at 200 rpm at 20 °C for 24 h. We conducted our experiments with solutions of higher Mn concentrations to evaluate the maximum effectiveness of the adsorbents at the removal of Mn. The detailed compositions of DMAPAAQ, DMAPAAQ + FeOOH, DMAA and DMAA + FeOOH are described in our previous studies [11,12,13]. As activated charcoal and silica gel are effective adsorbents, we used them to examine their adsorptive effectiveness and compared them with our results. Although the removal of Mn increases significantly with increase in temperature [18], we experimented at 20 °C to maintain natural conditions. Following this, a 30 mL sample was collected using a syringe. The amount of Mn remaining in the solution was measured by inductively coupled plasma mass spectrometry (ICP-MS). The amount of Mn adsorbed was measured using Equation (1).

Q = [(C_0_ − C_v_) * V]/m (1)

where Q is the amount of Mn adsorbed [mol/g], C_0_ is the initial concentration [mol/L], C_v_ is the equilibrium concentration [mol/L], V is the volume of the solution [L], and m is the mass of the adsorbent [g].

To investigate the pH-sensitivity, 20 mg of dried DMAPAAQ + FeOOH, activated charcoal, and silica gel were added to 40 mL of Mn solution. Additionally, HCl and NaOH were added to the solution to obtain different pH values in the range of 2–11. The solution was subsequently placed in a small beaker and stirred at 120 rpm at 20 °C for 24 h. Thereafter, 35 mL of the sample was collected using a syringe. The amount of arsenic remaining in the solution was measured using ICP-MS.

### 2.4. Fourier Transform Infrared Spectroscopy

FTIR spectroscopy (IR-Prestige 21, Shimadzu, Kyoto, Japan) was used to examine the changes in the surface functional groups of DMAPAAQ + FeOOH, DMAPAAQ, and γ-FeOOH following the adsorption of Mn. The concentration of the Mn solution, prepared using distilled water, was 500 mg/L. To adsorb Mn, 20 mg each of dried DMAPAAQ  +  FeOOH, DMAPAAQ, and γ-FeOOH were immersed in a 500 mg/L Mn solution for 24 h at 25 °C. Subsequently, the Mn-loaded gel pieces were carefully separated from the solutions and dried in an oven at 50 °C for 24 h. FTIR spectra were recorded in the wavelength range of 400–4000 cm^−1^ for DMAPAAQ, DMAPAAQ  +  FeOOH, γ-FeOOH, and their respective Mn-loaded samples. Potassium bromide (KBr)-pellet technique was used for FTIR analysis.

## 3. Results and Discussion

### 3.1. Adsorption of Mn Using the Cationic Gel and Its Composite

**Figure 1:**
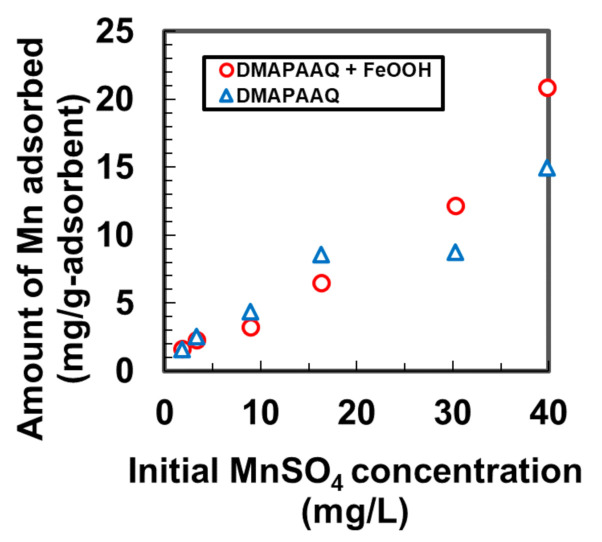
Amount of Mn adsorbed by DMAPAAQ and DMAPAAQ + FeOOH at different initial concentrations of MnSO_4_.

To examine the efficiency of Mn removal using the cationic gel and cationic gel composite, we experimented with the amount of adsorption of Mn using the DMAPAAQ + FeOOH and DMAPAAQ gels. Figure 1 shows that the amount adsorbed increased with the increase in the concentration of the Mn solution when DMAPAAQ + FeOOH was used. An identical trend was noted for DMAPAAQ. However, the adsorption amount of Mn was higher for DMAPAAQ + FeOOH than for DMAPAAQ at initial MnSO_4_ concentrations higher than 25 mg/L. The maximum amounts of Mn adsorbed by DMAPAAQ and DMAPAAQ + FeOOH were 13.32 and 39.02 mg/g, respectively. DMAPAAQ achieved its maximum adsorption amount of Mn following impregnation with γ-FeOOH particles. The DMAPAAQ + FeOOH gel composite contained 62.1% (*w*/*w*) of γ-FeOOH in its gel structure [13], which increased its Mn adsorption capacity.

Table 1 shows that the adsorption of Mn using DMAPAAQ + FeOOH gel follows the Langmuir isotherm closely, with an R^2^ of 0.92. Equation (2) shows the Langmuir model equation.

C_e_/Q_e_ = 1/(K_b_ Q_max_) + C_e_/Q_max_ (2)

where C_e_ is the concentration of adsorbate solution (mg/L) after adsorption equilibrium, Q_e_ is the adsorption capacity of the sample (mg/g dry adsorbent), K_b_ is the adsorption coefficient (L/mg), and Q_max_ is the maximum adsorption amount (mg/g).Therefore, ionic or covalent chemical bonds were formed between the adsorbent (DMAPAAQ + FeOOH) and the adsorbate (Mn) [19]. The adsorption sites provided by the particles of γ-FeOOH, in the structure of DMAPAAQ + FeOOH, form ionic bonds with Mn ions. Moreover, the amino groups of the DMAPAAQ polymer, in the structure of DMAPAAQ + FeOOH, bind with As ions. Hence, monolayer adsorption occurs in the structure of the gel composite, and multilayer adsorption was not possible.

On the other hand, in the case of DMAPAAQ gel, the methyl groups adhering to the amino group in the structure of DMAPAAQ bind with sulphate through ionic interactions. Sulphate ions additionally bind with Mn^2+^. Consequently, the cationic gel, DMAPAAQ, can adsorb Mn, despite Mn being a cation.

### 3.2. Comparative Adsorption of Mn

**Figure 2:**
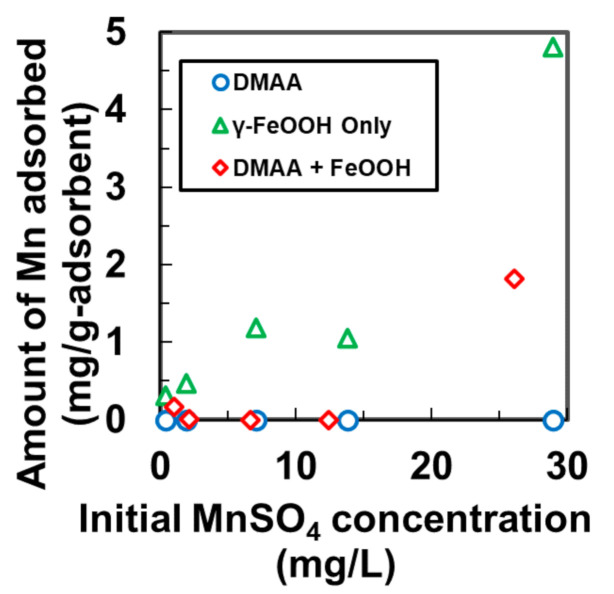
Amount of Mn adsorbed by DMAA, γ-FeOOH, and DMAA + FeOOH at different initial MnSO_4_ concentrations.

To further understand how γ-FeOOH increased the adsorption and to evaluate the advantage of the cationic gels over non-ionic gels, we experimented with the amount of Mn adsorbed using the non-cationic gel composite, DMAA + FeOOH, DMAA, and γ-FeOOH. Figure 2 shows that in the adsorption of Mn by the DMAA gel was nearly zero, which occurred because there was no bonding between the adsorption sites of the gel and the Mn ions. The amount of Mn adsorbed by DMAA + FeOOH was marginally higher than that of DMAA. Since DMAA cannot adsorb Mn, we infer that the impregnated FeOOH particles contributed to the adsorption of Mn by DMAA + FeOOH by bonding with Mn ions. However, γ-FeOOH achieved significant adsorption of Mn, which increased with the increase in the initial concentration of the MnSO_4_ solution.

The thermogravimetric analysis of DMAA + FeOOH suggests that the fraction of FeOOH in the gel was 51.3% (*w*/*w*). The adsorbed amounts of Mn by DMAA + FeOOH, γ-FeOOH, and DMAA gel were 0.78, 2.4, and 0 mg/g, respectively, when the concentration of MnSO_4_ was 26 mg/L. Therefore, the 51.3% (*w*/*w*) of γ-FeOOH particles were the sole contributor to DMAA + FeOOH being able to adsorb Mn. Similarly, γ-FeOOH particles improved the amount of adsorption of Mn in DMAPAAQ + FeOOH because we observed an increase in the Mn adsorption from 14.96 to 20.89 mg/g when DMAPAAQ was impregnated with γ-FeOOH. The presence of γ-FeOOH particles in the polymer structure helped remove contaminants because the latter adhered to the polymer binding sites [13]. However, at concentrations of Mn lower than 25 mg/L, the effectiveness of γ-FeOOH at improving the Mn adsorption amount was lower for DMAPAAQ + FeOOH and DMAA + FeOOH. At Mn concentrations lower than 25 mg/L, the Mn components did not strongly adhere to the adsorption sites of the polymer structure. Therefore, the removal of Mn under natural water conditions was possible when the cationic and non-ionic gels were impregnated with γ-FeOOH.

### 3.3. Simultaneous Adsorption of Mn and As

**Figure 3:**
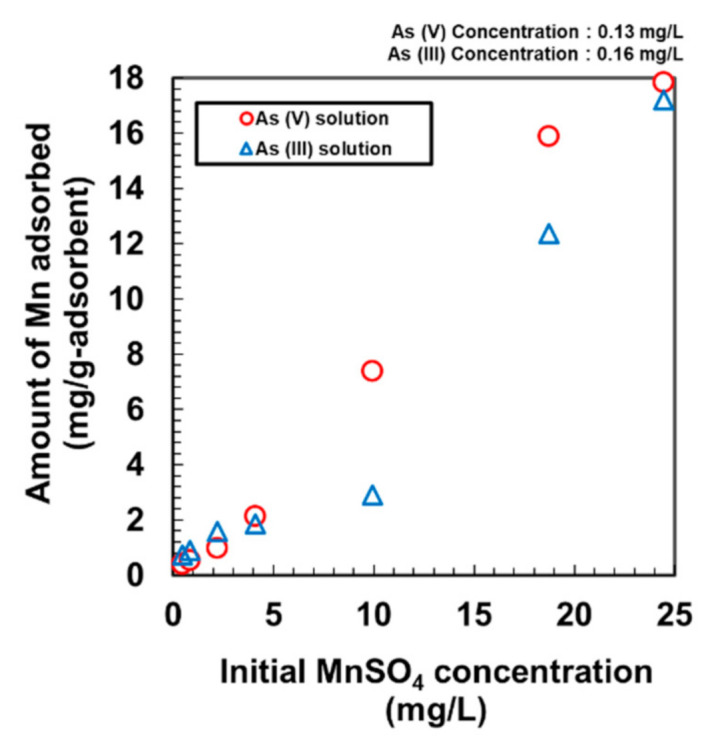
Adsorption of Mn by DMAPAAQ + FeOOH in the presence of As(III) and As(V) solutions.

**Figure 4:**
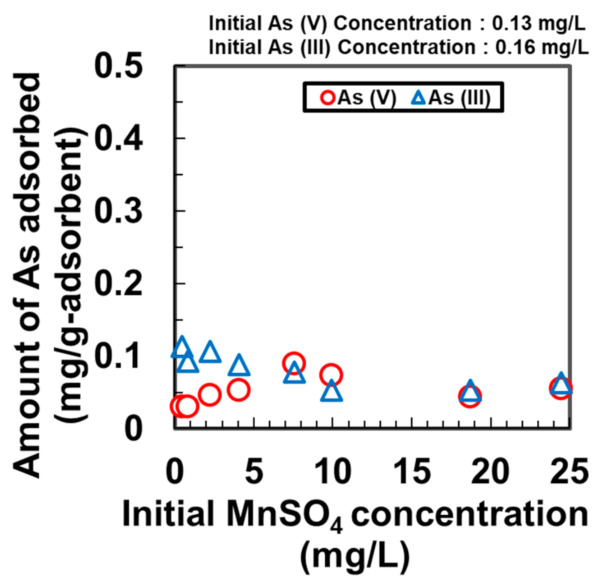
Adsorption of As(III) and As(V) by DMAPAAQ + FeOOH at different concentrations of Mn.

Previously, γ-FeOOH was proven to be effective at separately removing As [13,20,21] and Mn [14] discretely. However, the effectiveness of γ-FeOOH at simultaneously removing As and Mn has not been studied earlier. Additionally, the simultaneous removal of cations and anions by a single adsorbent has not been achieved previously. We examined the adsorption of Mn in the presence of As(V) and As(III) solutions (Figure 3), and the adsorption of As(V) and As(III) in the presence of Mn solution using DMAPAAQ + FeOOH (Figure 4).

In Figure 3, the adsorption of Mn from the solution of As(V) and As(III) is examined. The adsorbent was DMAPAAQ + FeOOH. The concentrations of As(III) and As(V) solutions were 0.16 and 0.13 mg/L, respectively. We conducted our experiments at low As concentrations because precipitation occurred at high As concentrations. Consequently, the actual amounts of Mn or As adsorbed could not be ascertained. However, at the As concentrations at which we conducted our experiment, no precipitation occurred. Figure 3 shows that the adsorption of Mn was nearly unaffected by the presence of As (Figure 1). A negligible increase in the adsorption of Mn in Figure 3 can be observed due to the forming of precipitation.

Figure 4 shows that the amounts of As(V) and As(III) adsorbed remained nearly constant despite the presence of Mn in the solution. Figure 4 suggests that when the initial concentration of MnSO_4_ was lower, the amount of adsorption of As(V) was similar to that from the samples where the initial amount of MnSO_4_ was higher. A similar phenomenon was observed when the adsorption of As(III) was studied in the presence of increasing Mn concentrations. The amount of As(III) adsorbed remained nearly constant despite the variation in the concentration of the Mn solution.

The two aforementioned observations from Figure 3 and Figure 4 suggest that the simultaneous adsorption of Mn and As by DMAPAAQ + FeOOH gel was achieved because As and Mn did not compete to adhere to the adsorption sites, which could accommodate both species simultaneously. The As ion was exchanged with the amino group in the polymer structure of DMAPAAQ, whereas Mn was connected to γ-FeOOH. An earlier report suggests that As can be adsorbed onto Mn but is subsequently re-adsorbed by Fe particles [22]. Hence, the adsorption sites provided by the γ-FeOOH particles in DMAPAAQ + FeOOH (which is 62.05% *w*/*w*) can adsorb As and Mn simultaneously.

The removal of As(V) was higher than that of As(III) at the initial MnSO_4_ concentrations of 7.56 and 9.95 mg/L. Removing As(III) is more difficult than removing As(V) [23]. At neutral pH, adsorption processes are ineffective on uncharged forms of As(III) [24], because As(III) cannot be ionised at neural pH. In contrast, As(V) is ionised at pH 7. These results prove that As and Mn can be effectively removed by DMAPAAQ + FeOOH.

### 3.4. Surface Functional Group Characterisation Using FTIR Spectroscopy

#### 3.4.1. FTIR Spectra of DMAPAAQ + FeOOH Gel Composite Following Mn Adsorption

**Figure 5:**
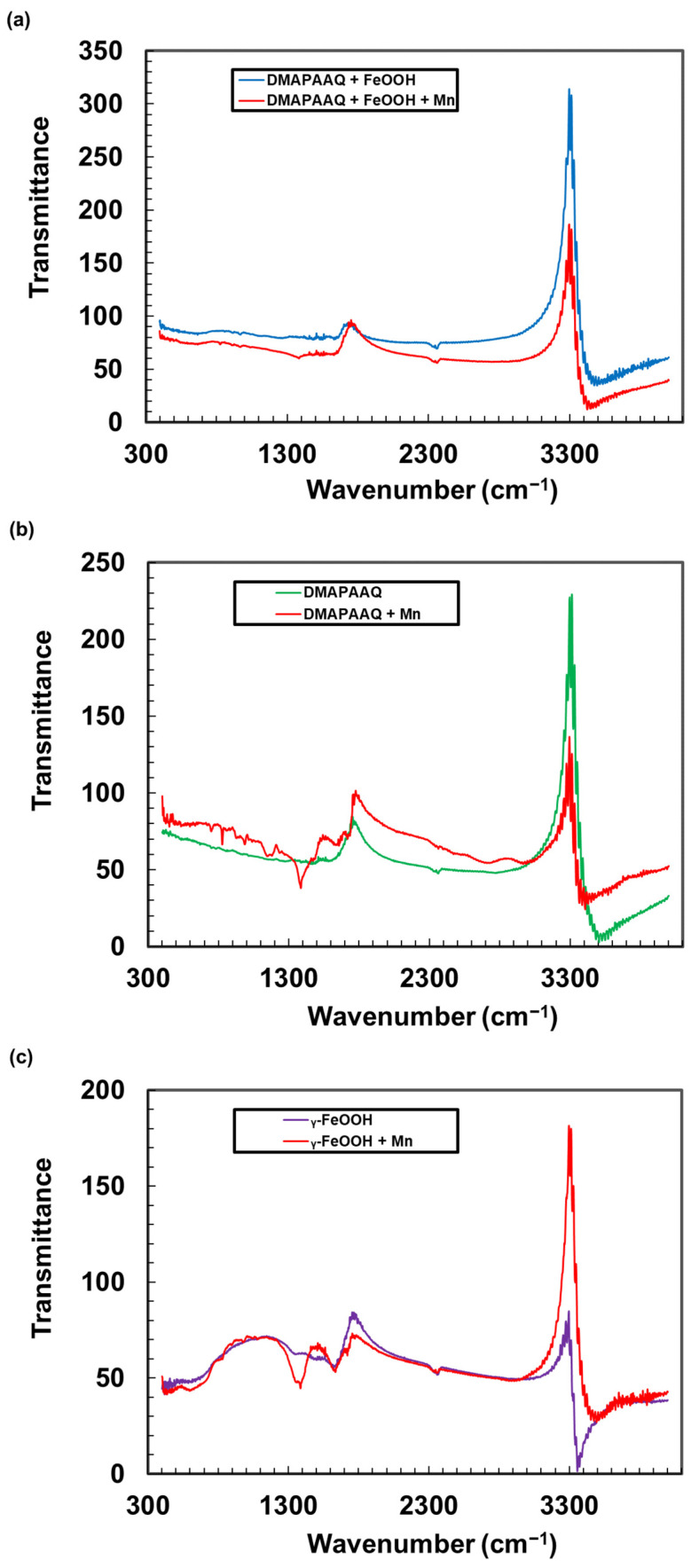
Comparative FTIR analysis of (**a**) DMAPAAQ + FeOOH and Mn-loaded DMAPAAQ + FeOOH, (**b**) DMAPAAQ and Mn-loaded DMAPAAQ, and (**c**) γ-FeOOH and Mn-loaded γ-FeOOH.

We compared the spectral peaks of the DMAPAAQ + FeOOH composites to determine the difference in the surface functional groups, before (blue line in Figure 5a) and after (red line in Figure 5a) the adsorption of Mn by the gel. As shown in Figure 5a and Table S1, in the FTIR spectrum of DMAPAAQ + FeOOH gel (blue line), there were shifts in the spectral bands, attributed to the vibrations of the alcohol O-H stretching, primary amine N-H stretching, aliphatic primary amine N-H stretching, secondary amine N-H amine stretching, and amine salt N-H stretching, respectively, following the adsorption of Mn by the DMAPAAQ + FeOOH gel composite. These shifts occurred due to the change in groups. In addition, the spectral peaks attributed to primary amine N-H stretching disappeared, and new peaks attributed to alcohol O-H stretching appeared in the spectral bands. The spectral bands attributed to secondary amine N-H stretching, alcohol O-H stretching, alkane C-H stretching, carbon dioxide O=C=O stretching, carboxylic acid C=O stretching, nitro compound N-O stretching and alkane C-H bending remained unchanged for DMAPAAQ + FeOOH.

#### 3.4.2. FTIR Spectra of DMAPAAQ Gel Following Mn Adsorption

The FTIR spectrum of DMAPAAQ was examined to compare the effect of γ-FeOOH on the adsorption of Mn. As Figure 5b and Table S1 show, in the FTIR spectrum of DMAPAAQ (red line), there were shifts in the spectral bands following the adsorption of Mn by the DMAPAAQ gel, ascribed to the vibrations of alcohol O-H stretching, primary amine N-H stretching, aliphatic primary amine N-H stretching, alcohol O-H stretching and amine C-N stretching, respectively. These shifts occurred due to the change in groups. In addition, the spectral peaks ascribed to alcohol O-H stretching and carboxylic acid C=O stretching disappeared, and new peaks appeared in the spectral bands ascribed to primary amine N-H stretching, alcohol O-H stretching and vinyl/phenyl ester C=O stretching. The spectral bands ascribed to secondary amine N-H stretching, alcohol O-H stretching, nitro compound N-H stretching, and alkane C-H bending remained unchanged for the DMAPAAQ gel.

#### 3.4.3. FTIR Spectra of DMAA + FeOOH Gel Composite Following Mn Adsorption

The FTIR spectrum of the non-ionic gel composite of DMAA + FeOOH gel composite was examined to compare the characteristics of the ionic and non-ionic gel composites. As shown in Figure 5c and Table S1, in the FTIR spectrum of DMAA + FeOOH (purple line), there was shift in the spectral band ascribed to the vibration of the alcohol O-H stretching following the adsorption of Mn by DMAA + FeOOH. This shift occurred because the group was altered. In addition, the spectral peaks ascribed to alcohol O-H stretching and alkane C-H stretching disappeared, and new peaks ascribed to alcohol O-H stretching, primary amine N-H stretching, aliphatic primary amine N-H stretching, and primary amide C=O stretching appeared in the spectral bands. The spectral bands ascribed to aliphatic primary amine N-H stretching, secondary amine N-H stretching, alcohol O-H stretching, alkyne C≡C Stretching, vinyl/phenyl ester C=O stretching, carboxylic acid C=O stretching, and alkane C-H bending remained unchanged for DMAA + FeOOH.

The above analysis suggests that the surface functional characteristics are altered following the loading of Mn into DMAPAAQ + FeOOH, DMAPAAQ gel and γ-FeOOH structure. Changes in the surface functional characteristics have also been reported when As(III) and As(V) were adsorbed by DMAPAAQ + FeOOH [11]. In addition, the cationic and non-ionic gel composites showed differences in the changes to the surface functional groups. These results prove that Mn was successfully adsorbed on the surface functional groups of the cationic gel, cationic gel composite, and anionic gel composite.

### 3.5. Effect of Experimental Parameters on the Adsorption of Mn by DMAPAAQ + FeOOH

**Figure 6:**
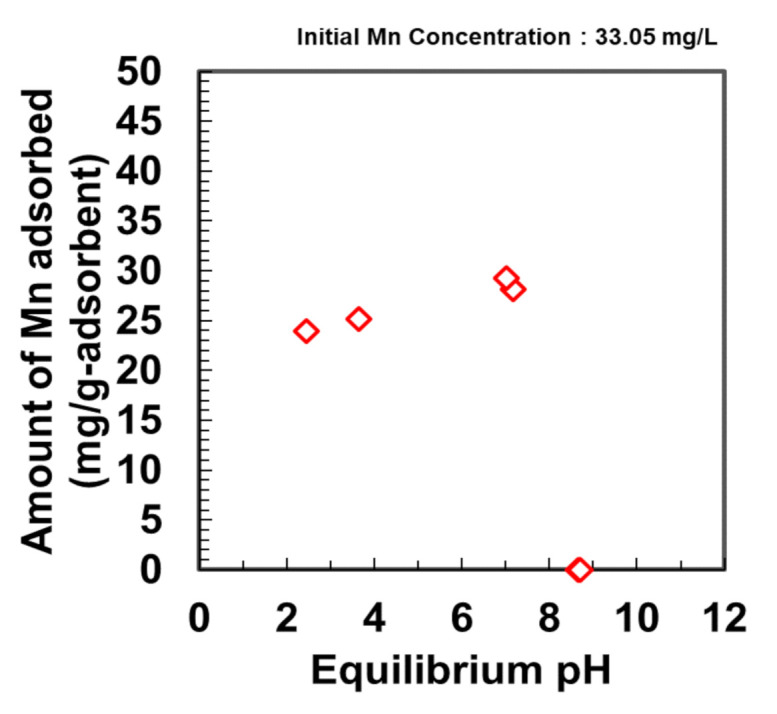
Effect of pH on the adsorption of Mn by DMAPAAQ + FeOOH gel.

The pH-sensitivity of Mn adsorption by DMAPAAQ + FeOOH was investigated using an Mn solution of 33.05 mg/L concentration. Different concentrations of HCl and NaOH were used in combination with the Mn solution to ascertain the arsenic adsorption levels at different pH. The samples were prepared by immersing 0.2 g of DMAPAAQ + FeOOH in various solutions having different pH ranging from 2 to 9. In all the samples, the initial concentration of Mn was 33.05 mg/L. The adsorption was performed at 20 °C under stirring at 194 rpm. Samples were collected following 24 h of adsorption. Figure 6 shows that the amount of adsorption increased slightly in the case of DMAPAAQ + FeOOH between pH 2 and 7. This increase in the adsorption of Mn was attributed to the surface of the DMAPAAQ + FeOOH gel composite becoming adsorption-friendly, which favoured the adsorption of the positively charged Mn [25]. The extent of adsorption was maximum at neutral pH, and it reduced as the pH of the solution increased. This might be attributed to the partial hydrolysis of Mn(II) ions with increasing pH, resulting in the formation of complexes with OH^−^, such as Mn(OH)^+^, Mn(OH)^2^, Mn_2_(OH)^3+^, Mn_2_OH^3+^, and Mn(OH)_4_^2−^ species in solution [26]. We surmise that Mn-hydroxyl species may participate in the adsorption and/or precipitation onto the adsorbent structure [7]. Zeolites have been reported to exhibit similar behaviour [27].

However, the amount of adsorption diminished at high pH. At higher pH levels, precipitation occurs because of the oxidation of Mn(II) [28]. A similar observation was reported when Mn was adsorbed by natural zeolitic tuff from the Vranjska Banja deposit in Serbia [29].

In addition, Budinova et al. (2009) [7] reported similar observations when Mn was adsorbed by bean pod carbon. The effect of pH on the removal of Mn(II) using bean pod carbon showed that the removal of manganese ionic species remained constant between pH 2.75 and 6.

This is a significant finding because, as discussed previously in the ‘Introduction’ section, most polymer gels and other adsorbents fail to adsorb Mn effectively at neutral pH. The maximum adsorption of Mn was obtained at pH 7.02 for DMAPAAQ+ FeOOH. Therefore, the remainder of the experiments were conducted at neutral pH, considering the real-life conditions of Mn-affected water.

### 3.6. Comparison to the Adsorption of Mn Using Other Adsorbents

**Figure 7:**
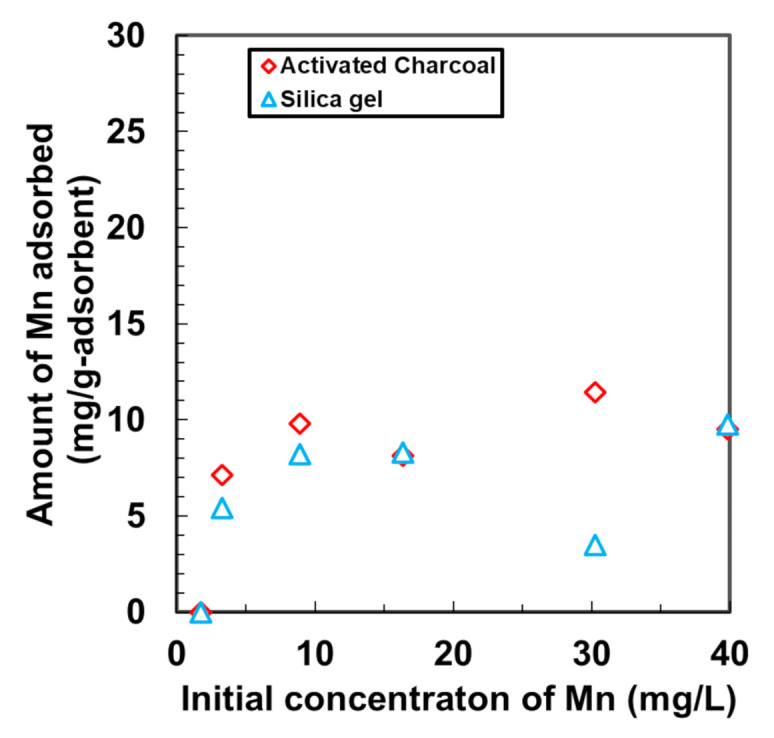
Adsorption of Mn by activated charcoal and silica gel.

We investigated the adsorptive removal of Mn using other adsorbents such as silica gel and activated charcoal. The samples were prepared by immersing 0.2 g of activated charcoal and silica gel in 40 mL of Mn solutions of different concentrations including 1, 2, 5, 10, 20, 25, and 40 mg/L. The adsorption was performed at 20 °C under stirring at 194 rpm. Following 24 h of adsorption, the samples were collected. The results shown in Figure 7 indicate that Mn was sparingly adsorbed by these two gels, and that the adsorption followed the Langmuir adsorption isotherm. At neutral pH, the maximum adsorption by the silica gel and activated charcoal were 6.89 and 10.82 mg/L, respectively, which are lower than that by DMAPAAQ + FeOOH at initial concentrations of Mn higher than 15 mg/L. Compared to the adsorption sites provided by activated charcoal and silica gel, the presence of γ-FeOOH in the structure of DMAPAAQ + FeOOH made the adsorption sites more favourable for Mn to adhere to and be adsorbed.

In addition, other adsorbents previously studied for the removal of Mn had less maximum adsorption capacity than that of DMAPAAQ + FeOOH. The maximum adsorption amount of Mn by the other adsorbents, for instance, granular activated carbon [30], clinoptilolite from Turkey [31], quartz [32], marble [32], dolomite [32], Na-montmorillonite [33], clinoptilolite-Fe oxide [34], tannic acid immobilised activated carbon [35], natural sepiolite [36], natural zeolite (NZ) [37], and activated carbon from bean pods waste [7] were 2.54, 4.22, 0.06, 1.20, 2.21, 3.22, 27.12, 1.73, 0.25, 7.68, and 23.4 mg/g, respectively. Although these maximum adsorption amount values followed different experimental conditions and no experimental relation between them existed, these adsorbents only represent the Mn retention tendency by the respective adsorbents.

## 4. Conclusions

In summary, Mn was effectively removed by the DMAPAAQ gel, DMAPAAQ + FeOOH gel composite, DMAA + FeOOH gel composite, activated charcoal, silica gel, and γ-FeOOH at natural water conditions. The highest amount of Mn adsorption was achieved by DMAPAAQ + FeOOH at 39.02 mg/g. This occurred because the presence of γ-FeOOH in the polymer structure of DMAPAAQ + FeOOH helped to increase its efficiency at removing Mn, similar to how γ-FeOOH improved the Mn-adsorption performance of DMAA + FeOOH. Therefore, the DMAPAAQ + FeOOH gel removes Mn more effectively than the abovementioned adsorbents.

This was a pioneering study on the simultaneous adsorption of As and Mn. We concluded that As and Mn can be removed simultaneously using DMAPAAQ + FeOOH without impacting the removal performance, because As was attached to the amino group in the polymer structure of DMAPAAQ as AsO_7_^−^ and Mn was attached to γ-FeOOH. The uptake of As(V) was greater than that of As(III) because As(III) cannot be ionised at neutral pH, though As(V) can. Additionally, pH had no effect on the adsorption of Mn by DMAPAAQ + FeOOH at neutral and acidic pH. The maximum adsorption of Mn by DMAPAAQ + FeOOH was attained at pH 7.02, which is an important finding for the practical implementation of the adsorbent in removing Mn. Finally, the FTIR results prove that Mn was successfully attached to the surface functional groups of DMAPAAQ + FeOOH. No other work on the removal of Mn has performed FTIR analysis to date. Further study is required in the future, since these results need to be compared to those of anionic gels and anionic gel composites and other green polymeric adsorbents. Our aim is to simultaneously remove harmful metal cations such as those of As, Mn, Cr, and Cd, as well as other components.

## Figures and Tables

**Table 1:** Adsorption isotherm parameters for the adsorption of Mn and As on DMAPAAQ + FeOOH.

Isotherm Parameter		Mn	As
Langmuir model	R^2^	0.92	0.997
	Q_max_, mg/g	39.02	123.4

## Data Availability

The data presented in this study are available in article or Supplementary Material..

## Supplementary Materials

The following are available online at https://www.mdpi.com/article/10.3390/nano11041032/s1, Table S1: FTIR spectroscopy peak analysis, Table S2: Composition of gel composite, Table S3: Composition of gel.
