# Supplementary material for: Simultaneous Removal of Arsenic and Manganese from Synthetic Aqueous Solutions Using Polymer Gel Composites
Source: Nanomaterials (Basel). 2021 Apr 18;11(4):1032. doi: 10.3390/nano11041032 (PMC8073374; doi:10.3390/nano11041032)
Supplement: Supplementary file 1 [file nanomaterials-11-01032-s001.zip › nanomaterials-1158500-supplementary.pdf]

nanomaterials

Supporting Information

Simultaneous Removal of Arsenic and Manganese from Synthetic Aqueous Solutions Using Polymer Gel Composites

Syed Ragib Safi and Takehiko Gotoh *

Department of Chemical Engineering, Hiroshima University, 1-4-1 Kagamiyama, Higashi Hiroshima, Hiroshima 739-8527, Japan; d196289@hiroshima-u.ac.jp
* Correspondence: tgoto@hiroshima-u.ac.jp; Tel.: +81-82-424-7720

Table S1. FTIR spectroscopy peak analysis.

Wavelength	DMAPAAQ + FeOOH	DMAPAAQ + FeOOH + Mn	DMAPAAQ	DMAPAAQ + Mn	γ-FeOOH	γ-FeOOH + Mn	Group	Compound Class	Appearance
1209							C-N stretching	amine	medium
1211									
1458							C-H bending	alkane	medium
1508							N-O stretching	nitro compound	strong
1541									
1693							C=O stretching	primary amide	strong
1753							C=O stretching	carboxylic acid	strong
1764							C=O stretching	vinyl/phenyl ester	strong
1774									
2139							C≡C Stretching	alkyne	weak
2347							O=C=O stretching	carbon dioxide	strong
2387									
2954							N-H stretching	amine salt	strong, broad
2956									
3047							C-H stretching	alkane	medium
3051									
3234							O-H stretching	alcohol	strong, broad
3255									
3273									
3275									
3294									
3296									

3311							N-H stretching	second-ary amine	medium
3329									
3331									
3346									
3348									
3365							N-H stretching	ali-phatic primary amine	medium
3367									
3369									
3373									
3385									
3387									
3390									
3404							N-H stretching	primary amine	medium
3412									
3415									
3423									
3433									
3435									
3442									
3448									
3456									
3458									
3464									
3469									
3473									
3475									
3481									
3483									
3489									
3500									
3504									
3523									
3535									
3539									
3547									
3618							O-H stretching	alcohol	me-dium, sharp
3620									
3641									
3649									
3651									
3660									
3668									
3674									

3676			
3678			

Table S2. Composition of gel composite.

	Chemical	Quantity (mol/m³)
**Monomer**	DMAPAAQ, DMAA	500
**Crosslinker**	MBAA	50
**Accelerator**	Sodium Sulfite	80
	Sodium Hydroxide (NaOH)	2100
**Initiator**	Ammonium peroxodisulfate (APS)	30
	Ferric Chloride ($FeCl_3$)	700

Table S3. Composition of gel.

.	Chemical	Quantity (mol/m³)
**Monomer**	DMAPAAQ, DMAA	1000
**Crosslinker**	MBAA	50
**Accelerator**	Sodium Sulfite	20
**Initiator**	Ammonium peroxodisulfate (APS)	5
